# Genome Sequence of *Nitrosomonas communis* Strain Nm2, a Mesophilic Ammonia-Oxidizing Bacterium Isolated from Mediterranean Soil

**DOI:** 10.1128/genomeA.01541-15

**Published:** 2016-01-14

**Authors:** Jessica A. Kozlowski, K. Dimitri Kits, Lisa Y. Stein

**Affiliations:** Department of Biological Sciences, University of Alberta, Edmonton, Alberta, Canada

## Abstract

The complete genome sequence of *Nitrosomonas communis* strain Nm2, a mesophilic betaproteobacterial ammonia oxidizer isolated from Mediterranean soils in Corfu, Greece, is reported here. This is the first genome to describe a cluster 8 *Nitrosomonas* species and represents an ammonia-oxidizing bacterium commonly found in terrestrial ecosystems.

## GENOME ANNOUNCEMENT

The isolation and study of ammonia-oxidizing bacteria (AOB) are essential to furthering our understanding of their role in nitrogen cycling across diverse environments, and particularly their contribution to nitrate pollution and nitrous oxide (N_2_O) emissions. The AOB *Nitrosomonas communis* Nm2 was first isolated from Corfu, Greece (1), and is a mesophilic aerobic betaproteobacterium belonging to *Nitrosomonas* cluster 8 (2).

The genome of *N. communis* was sequenced at the University of Washington, WA, using the PacBio RSII platform; 150,292 raw reads resulted in 110,554 quality-filtered trimmed reads yielding 429.2 Mb, with a mean genome-wide coverage of 96×. The filtered reads were assembled at the University of Alberta, Alberta, Canada, using HGAP version 2.3 (3), resulting in a 1-contig scaffold. Annotation was performed using the NCBI Prokaryotic Genome Annotation Pipeline (4). The genome is 4.07 Mbp, with a mean G+C content of 44.73%, and it contains 3,189 predicted protein-coding genes. The genome includes 41 tRNA genes and a single copy of the 16S-23S-5S rRNA operon. Gene prediction analysis and comparative genomics were performed with IMG (5). The closest neighbor of *N. communis* was *Nitrosomonas* sp. Is79 (6), with an average nucleotide identity (ANI) (7) of 72.02%.

*N. communis* oxidizes ammonia to nitrite as a sole source of energy and reductant. The genome contains 2 operons for ammonia monooxygenase (*amoCAB*), one of which is followed by genes for copper transport and resistance proteins, *copCD* (8, 9). Three operons for hydroxylamine dehydrogenase (*haoAB-cycAB*) were found, one of which lacked a copy of *cycB*, a feature found in both *Nitrosomonas europaea* and *Nitrosomonas eutropha* (8, 9). A single copy of the AOB-specific red-copper protein nitrosocyanin (10) was also found. Although genes for a complete urease are present in some *Nitrosospira* genomes (11, 12), *N. communis* encodes only the urea carboxylase (EC 6.3.4.6) and putative allophanate hydrolase (EC 3.5.1.54), similar to inventory found in the oligotrophic *Nitrosomonas* sp. Is79 (6, 13). Genes for carbon fixation, including two copies of RubisCO-encoding genes, were identified with similarity to those found in *Nitrosomonas* sp. Is79 (6). AOB living in terrestrial ecosystems can experience high nitrogen loads and varied oxygen tensions, conditions connected with increased production of nitrous oxide. Inventory for nitrifier denitrification was identified in *N. communis*; two nitric oxide reductases (*norCBQD* and *norSY-senC-orf1*), two copies of the NO-responsive regulator *NnrS*, cytochrome P460 (*cytL*), and cytochrome *c*′-beta (14). Although the majority of AOB genomes contain *nirK* (15), the gene for the copper-containing nitrite reductase, no homologues were found in *N. communis* for either *nirK* or the cytochrome *cd*_1_ *nirS* nitrite reductases; however, a recent study showed that NirK is not required for nitrifier denitrification in *N. europaea* (16).

Genes for nickel-iron-containing, NAD-reducing, hydrogen dehydrogenase were identified, with the exception of *hoxH* (11). For iron acquisition and storage, *N. communis* contains one set of genes for a *Streptococcus*-like ferric iron ABC transporter (8), three copies of TonB-associated ferric siderophore transporters (17), genes for siderophore synthesis and export, and three copies of bacterioferritin and associated genes for intracellular iron storage.

### Nucleotide sequence accession number.

The genome sequence has been deposited in GenBank under the accession number CP011451. The version described in this paper is the first version.
